# Engagement of Neurotropic Viruses in Fast Axonal Transport: Mechanisms, Potential Role of Host Kinases and Implications for Neuronal Dysfunction

**DOI:** 10.3389/fncel.2021.684762

**Published:** 2021-06-21

**Authors:** Alexsia Richards, Sarah H. Berth, Scott Brady, Gerardo Morfini

**Affiliations:** ^1^Whitehead Institute for Biomedical Research, Cambridge, MA, United States; ^2^Department of Neurology, Johns Hopkins School of Medicine, Baltimore, MD, United States; ^3^Department of Anatomy and Cell Biology, University of Illinois at Chicago, Chicago, IL, United States

**Keywords:** neurotropic virus, axonal transport, kinesin, dynein, kinase, herpes simplex virus, rabies virus

## Abstract

Much remains unknown about mechanisms sustaining the various stages in the life cycle of neurotropic viruses. An understanding of those mechanisms operating before their replication and propagation could advance the development of effective anti-viral strategies. Here, we review our current knowledge of strategies used by neurotropic viruses to undergo bidirectional movement along axons. We discuss how the invasion strategies used by specific viruses might influence their mode of interaction with selected components of the host’s fast axonal transport (FAT) machinery, including specialized membrane-bounded organelles and microtubule-based motor proteins. As part of this discussion, we provide a critical evaluation of various reported interactions among viral and motor proteins and highlight limitations of some *in vitro* approaches that led to their identification. Based on a large body of evidence documenting activation of host kinases by neurotropic viruses, and on recent work revealing regulation of FAT through phosphorylation-based mechanisms, we posit a potential role of host kinases on the engagement of viruses in retrograde FAT. Finally, we briefly describe recent evidence linking aberrant activation of kinase pathways to deficits in FAT and neuronal degeneration in the context of human neurodegenerative diseases. Based on these findings, we speculate that neurotoxicity elicited by viral infection may involve deregulation of host kinases involved in the regulation of FAT and other cellular processes sustaining neuronal function and survival.

## Introduction

The large size and highly polarized cellular architecture of neurons present a major challenge for the replication and propagation of neurotropic viruses. This is because the cellular machinery typically needed for their replication is located in the neuronal soma, far away from axonal terminals where neurotropic viruses typically invade neurons (Salinas et al., 2010). Depending on the specific neuronal cell type, neurotropic viruses need to move along axons for distances up to a meter or longer before reaching the somatic compartment. Conversely, newly synthesized virions often need to undergo transport from the neuronal cell soma back to axonal termini for eventual release and propagation. Some neurotropic viruses undergo trans-synaptic spread among connected neurons. This form of transmission involves the transport of viruses from cell soma to presynaptic terminals of one neuron, and from dendritic post-synapses to somata of target neurons (Beier, 2019). Thus, the movement of neurotropic viruses between neuronal subcompartments represents an essential aspect of their life cycle.

The complex composition of the axoplasmic milieu, the remarkable length of axons, and the large size of most neurotropic viruses prevent these viruses from reaching their final destinations through mere diffusion (Sodeik, 2000; Leopold and Pfister, 2006). To move along axons, viruses need to engage in *fast axonal transport* (FAT), a cellular process involving the intracellular trafficking of membrane-bounded cellular organelles (MBOs) by microtubule-based motor proteins (Black, 2016). An understanding of mechanisms by which neurotropic viruses exploit their host FAT machinery could potentially facilitate the development of antiviral strategies targeting this task, as this process is critically needed to complete major stages of the viral life cycle. In addition, and given the heavy reliance of neurons on FAT, such an understanding may also provide a molecular basis underlying the dysfunction and degeneration of these cells commonly associated with a viral infection.

In this review, we provide a succinct description of mechanisms used by some neurotropic viruses to engage in FAT. While numerous reviews have covered such mechanisms in detail (Sodeik, 2000; Diefenbach et al., 2008; Salinas et al., 2010; Zaichick et al., 2011), ours brings into consideration a significant body of recent experimental work revealing a role of various protein kinases on the regulation of FAT, often through direct phosphorylation of motor proteins powering this cellular process (Gibbs et al., 2015; Brady and Morfini, 2017). Based on this evidence, we speculate that localized activation of host kinases may play a role in the initial engagement of some virions in retrograde FAT. Focusing on specific members of the Alphaherpeviridae, Flaviviridae, Rhabdoviridae, and Picornaviridae viral families, we describe how well-established strategies used by these viruses to invade neurons might set the stage for interactions with specific components of the host FAT machinery, including endocytic vesicular organelles and motors proteins. Within this context, we provide a critical evaluation of prior work reporting various interactions between viral proteins and major microtubule-based motor proteins powering FAT (Berth et al., 2009). Our evaluation takes into consideration the multi-subunit composition and biochemical heterogeneity of these motors, an issue rarely mentioned in the published literature (Brill and Pfister, 2000; Morfini et al., 2016). Finally, we describe evidence linking aberrant activation of kinases to FAT deficits in human neurological diseases (Brady and Morfini, 2017), as well as work documenting deregulation of neuronal kinases in association with a viral infection. Based on this premise, we hypothesize that some pathological features of neurons infected by neurotropic viruses may result, at least in part, from aberrant modulation of kinases involved in the regulation of FAT and other cellular processes sustaining neuronal function and survival.

## Fast Axonal Transport, A Cellular Process Powered by Microtubule-Based Motor Proteins

**Figure 1:**
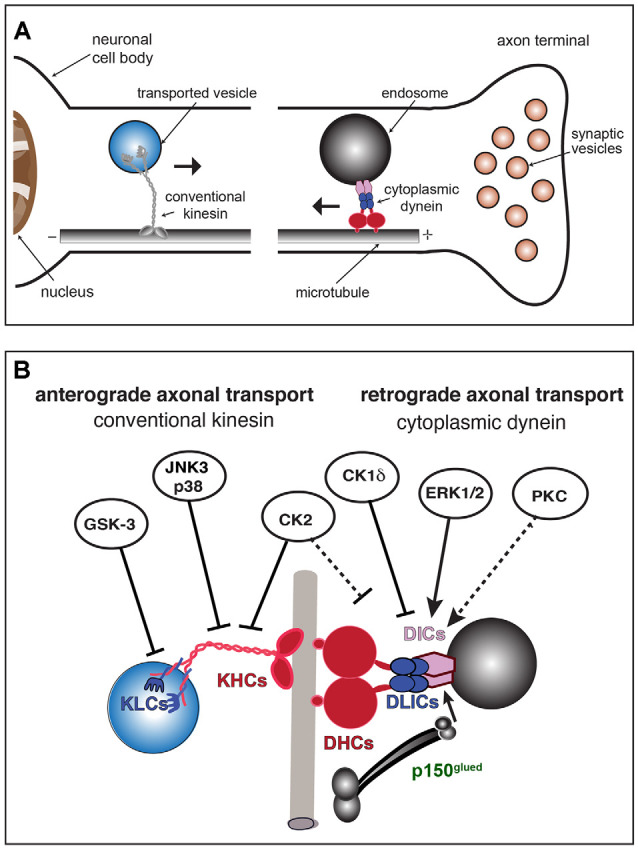
Conventional kinesin and cytoplasmic dynein (CDyn): Transport of cargo, subunit components, and regulation by protein kinases. **(A)** Anterograde fast axonal transport (FAT; represented by →) of membrane-bounded organelles (MBOs) is carried out by plus-end directed microtubule-based motors of the kinesin superfamily. Among those, conventional kinesin is the most abundant in mature neurons. Conventional kinesin moves a wide variety of MBOs from their location of synthesis and packaging in the neuronal soma to spatially discrete axonal subcompartments, including presynaptic terminals. The minus-end directed motor, cytoplasmic dynein, drives retrograde FAT of MBOs containing degraded materials, defective organelles, or signaling endosomes back to the neuronal soma (←). **(B)** Subunit organization of major microtubule-based motors. The conventional kinesin holoenzyme (left) is a heterotetramer composed of two kinesin heavy chain (KHC) and two light chain (KLC) homodimers. Cytoplasmic dynein (right) exists as a multisubunit protein complex formed by at least two heavy chains (DHCs, in red), two intermediate chains (DICs, in purple), four light intermediate chains (LICs, in blue), and several light chains (LCs, not shown). The dynactin subunit P150^Glued^ (in green) interacts with both DICs and microtubules and has been proposed to modulate the processive movement of the CDyn complex. Kinases with established roles in the regulation of anterograde and retrograde FAT are indicated. Solid arrows indicate specific motor protein subunits targeted by each kinase. Whether PKC activates retrograde FAT through cytoplasmic dynein phosphorylation has not yet been established (dashed arrow). Straight arrows indicate activation of retrograde FAT (by ERK1/2 and PKC). Blunt-ended arrows indicate inhibitory effects of kinases on either anterograde or retrograde FAT. By altering the activity of kinases involved in the regulation of FAT and motor protein phosphorylation, neurotropic viruses may promote FAT abnormalities and other cellular processes, eventually triggering neuronal dysfunction and pathology.

Microtubules are intrinsically polarized tubulin polymers, with alpha-tubulin exposed at their minus-end and beta-tubulin at their plus-end (Baas et al., 2016). Major cellular processes sustaining neuronal function and survival including the maintenance of axons, synaptic transmission, and trophic-dependent support all depend on the regulated trafficking of MBOs along microtubules. This process, referred to as *fast axonal transport* (FAT), is powered by motor proteins that convert energy derived from ATP hydrolysis into mechanical forces [reviewed in (Morfini et al., 2012; Black, 2016)]. Within axons, the uniform organization of microtubules allows FAT to proceed in either *anterograde* or *retrograde* directions (away and towards the neuronal soma, respectively; Figure 1A). In mature neurons, *conventional kinesin* and *cytoplasmic dynein* (CDyn) are the major microtubule-based motor proteins powering anterograde and retrograde FAT, respectively (Morfini et al., 2012; Hirokawa and Noda, 2008). Anterograde FAT involves the translocation and delivery of a wide variety of MBOs from their place of synthesis and packaging in the neuronal soma to spatially discrete axonal subcompartments including the axonal initial segment, pre-synaptic terminals, and nodes of Ranvier. Conversely, retrograde FAT involves the movement of specialized MBOs containing degraded materials, defective organelles, or neurotrophic signals from these axonal subdomains back to the neuronal soma. FAT is regarded as an essential cellular process sustaining the unique cytoarchitecture and connectivity of neurons (Morfini et al., 2012; Black, 2016). Accordingly, strong genetic and experimental evidence have highlighted the unique vulnerability of neurons to deficits in FAT (reviewed in Roy et al., 2005; Brady and Morfini, 2017; Sleigh et al., 2019).

As no viral genomes have been found to encode microtubule-based motor proteins to date, neurotropic viruses must somehow co-opt one or more components of the host’s FAT machinery to move from their initial site of neuronal entry (typically axonal termini) to their site of replication in the nucleus or perinuclear cytoplasm. Following replication, nascent virions can either engage in anterograde axonal transport and move towards the periphery or undergo sorting to dendrites and spread to post-synaptic neurons (Koyuncu et al., 2013). Little is known about mechanisms driving dendritic sorting of viral particles or how they are transported to this compartment, where microtubules have a mixed orientation (Conde and Cáceres, 2009). Below, we provide a brief overview of CDyn and conventional kinesin, major motor proteins powering anterograde and retrograde FAT, also highlighting the recent discovery of phosphorylation-based mechanisms for the regulation of this cellular process (Gibbs et al., 2015; Brady and Morfini, 2017).

### Cytoplasmic Dynein (CDyn)

CDyn drives retrograde FAT of mitochondria and various specialized MBOs including multivesicular bodies containing degraded cellular materials, autophagosomes carrying defective organelles, and signaling endosomes containing neurotrophins, which activate signaling pathways that promote gene transcription and survival upon arrival to the neuronal soma (Maday, 2016; Zahavi et al., 2017; Reck-Peterson et al., 2018). In addition, a significant fraction of the total CDyn cellular pool is not associated with membranes. Instead, this CDyn pool associates with components of the subcortical actin cytoskeleton to power the transport of short microtubules along axons in the anterograde direction (Rao et al., 2017).

*In vivo*, CDyn exists as a large multi-subunit protein complex of approximately 1.5 MDa (Brill and Pfister, 2000; Figure 1B, right). The native CDyn holoenzyme is composed of two dynein heavy chain subunits (DHCs), two dynein intermediate chain subunits (DICs), four dynein light intermediate chain subunits (DLICs), and a variable number of dynein light chain subunits (DLCs). Another multisubunit protein complex termed *dynactin* associates with a small fraction of total CDyn in cells (King and Schroer, 2000; McKenney et al., 2014). The largest protein subunit of the dynactin complex, p150^Glued^, has been proposed to regulate the initiation of retrograde FAT at the axonal terminal through interactions between its CAP-Gly domain and the plus end of microtubules (Lloyd et al., 2012).

DHC subunits, which contain domains responsible for binding to microtubules and ATP hydrolysis, confer upon CDyn its mechanochemical properties (Koonce, 1997). Unlike DHCs, our knowledge of specific functional roles played by DLIC and DLC subunits remains scant. DLC subunits, which directly bind to DICs as homodimers, include the proteins LC7, LC8, and Tctex (Williams et al., 2007). Based on their interactions with a wide variety of structurally unrelated proteins, these DLC subunits were initially proposed to act as adaptors linking the CDyn complex to specific MBO cargoes. However, structural data has cast doubts on this hypothesis, suggesting instead that they might function as hub proteins to promote assembly of the CDyn holoenzyme (Benison et al., 2007; Williams et al., 2007; Barbar, 2008).

Over recent years, an increasingly growing body of experimental evidence accumulated suggesting that DIC subunits mediate binding of CDyn holoenzymes to selected MBOs (reviewed in Canty and Yildiz, 2020). The wide heterogeneity of DIC isoform variants appears consistent with such a role. Specifically, two genes encoding DIC subunits are expressed in mammals (DIC1 and DIC2), which undergo extensive alternative splicing to produce a large number of isoforms displaying unique tissue distribution patterns and association with selected MBOs (Kuta et al., 2010). For example, the neuron-specific DIC1B isoform, but not the ubiquitously expressed DIC2C isoform, was found to colocalize with signaling endosomes containing activated neurotrophin receptors (Trks; Ha et al., 2008). Taken together, these and other observations suggest that unique CDyn holoenzyme variants, defined by their subunit isoform composition, associate with specific MBOs (Pfister, 2015). Unfortunately, the full complement of such variants in specific cell types remains unknown (Kuta et al., 2010). As discussed in “Engagement of Virions With Specific Components of the Host’s Fast Axonal Transport Machinery” section, this gap in our knowledge limits an assessment of the physiological relevance of interactions between viral proteins and specific CDyn subunits reported in the literature.

### Conventional Kinesin

Anterograde FAT is powered by the kinesin superfamily of motor proteins (reviewed in Hirokawa et al., 2009; Morfini et al., 2012). Of the multiple kinesin-related proteins expressed in the mature mammalian brain, *conventional kinesin* (kinesin-1) is by far the most abundant (Wagner et al., 1989; Morfini et al., 2012). Conventional kinesin mediates anterograde FAT of a wide variety of MBOs, including mitochondria and vesicular organelles containing unique sets of protein cargoes. *in vivo*, conventional kinesin exists as a multi-subunit protein complex composed of two heavy chain (KHC or KIF5) and two light chain (KLCs) subunit dimers (reviewed in Morfini et al., 2016; Figure 1B, left). KHC subunits confer mechanochemical properties to the conventional kinesin holoenzyme, containing both ATPase and microtubule-binding domains (Bloom et al., 1988). KLC subunits feature heptad repeats at the amino terminus, which mediate interactions with KHC subunits, and DnaJ-like domains at their central region that tightly associate to MBOs (Stenoien and Brady, 1997; Tsai et al., 2000).

Three genes encoding KHC subunits (*KIF5A*, *KIF5B*, and *KIF5C*) and two genes encoding KLC subunits (*KLC1*, and *KLC2*) are expressed in mammalian nerve tissues (Rahman et al., 1998; Kanai et al., 2000). Heavy and light chain subunit isoforms feature an overlapping yet unique pattern of expression, strongly suggesting cell-type-specific specializations in FAT (Kanai et al., 2000). KHCs and KLCs are organized as homodimers within the conventional kinesin holoenzyme (DeBoer et al., 2008). At least six different biochemical variants of conventional kinesin have been identified based on their subunit isoform composition, but many more may exist as a result of alternative *KLC1* gene splicing (Cyr et al., 1991; McCart et al., 2003). Additional experiments indicate that the unique carboxy termini of both KHC and KLC subunits play a role in the targeting of these variants to specific MBOs (Stenoien and Brady, 1997; Woźniak and Allan, 2006; DeBoer et al., 2008).

### Regulation of Fast Axonal Transport by Protein Kinases

Phosphoregulation of CDyn and conventional kinesin was first suggested by metabolic labeling experiments, which revealed differential phosphorylation of specific subunits *in vivo* (Dillman and Pfister, 1994; Elluru et al., 1995; Susalka and Pfister, 2000). Accordingly, several protein kinases were subsequently found to modulate anterograde and/or retrograde FAT (Figure 1B). Many of these findings stemmed from experiments in the isolated squid axoplasm preparation, a model system uniquely suited for the study of cellular processes operating in the axonal compartment (Kang et al., 2016; Song et al., 2016). A mechanistic basis for the effect of some protein kinases on FAT remains unknown, but several have been shown to impact specific functional activities of CDyn and conventional kinesin (i.e., binding to MBO cargoes or microtubules) through direct phosphorylation of specific subunits (reviewed in Gibbs et al., 2015; Morfini et al., 2009a). In addition, some kinases have been shown to impact FAT indirectly. For example, the protein kinase CDK5 was shown to modulate anterograde FAT through a PP1-GSK3 pathway (Morfini et al., 2004). Similarly, Akt and other kinases have been proposed to modulate FAT by targeting putative adaptor proteins linking motors to MBOs (Zala et al., 2008; Fu and Holzbaur, 2014). Collectively, findings of FAT regulation by protein kinases provided a potential mechanistic basis for the localized delivery of selected MBOs at discrete axonal subcompartments (Morfini et al., 2016). A succinct description of experimental work linking specific protein kinases to the regulation of FAT is provided below.

Several independent studies revealed specific kinases affecting CDyn-based retrograde FAT in various cell types (Figure 1B, right). For example, phosphorylation of DIC1B subunit isoform by ERK1/2 kinases was linked to the retrograde FAT of signaling endosomes in cultured neurons (Mitchell et al., 2012). In Xenopus melanophore cells, casein kinase 1δ (CK1δ) was found to activate retrograde transport of pigment granules by phosphorylating DIC subunits (Ikeda et al., 2011). Independently, experiments using the squid axoplasm preparation linked activation of selected protein kinase C (PKC) isoforms, including PKCδ, to increased retrograde FAT of synaptic vesicles (Morfini et al., 2007). Conversely, the protein kinases p38β (Morfini et al., 2013), casein kinase 2 (CK2; Morfini et al., 2001), and cJun-amino terminal kinase 3 (JNK3; Morfini et al., 2009b) were all found to inhibit retrograde FAT in this model, but whether this inhibitory effect involves direct phosphorylation of specific CDyn subunits has not yet been established (Pfister et al., 1996; Morfini et al., 2001).

Some kinases involved in the regulation of anterograde FAT have also been identified (Figure 1B, left). For example, JNK3 and p38α directly phosphorylate KHC subunits, negatively affecting the interaction of conventional kinesin with axonal microtubules that enables its processive movement (Morfini et al., 2009b, 2013). In addition, glycogen synthase kinase 3 (GSK-3) was found to directly phosphorylate KLC subunits, promoting detachment of conventional kinesin from MBOs through a chaperone-mediated process (Morfini et al., 2002). Finally, CK2 was found to inhibit anterograde FAT through a mechanism involving phosphorylation of both KHC and KLC subunits (Donelan et al., 2002; Zamponi et al., 2017).

Relevant to the main topic discussed in this review, numerous host kinases undergo abnormal activation during viral infection of neurons (McLean and Bachenheimer, 1999; Zachos et al., 1999; Nakamichi et al., 2005; Gillis et al., 2009; Gupta et al., 2011; Wang et al., 2015), including some involved in the regulation of motor proteins. As discussed in “Entry Routes Shape the Mode of Neurotropic Virus Engagement in FAT” section, this observation raises the possibility that localized activation of kinases at entry sites might promote the retrograde FAT of virions. By extension, it is also conceivable that aberrant kinase signaling triggered by viruses could negatively impact the homeostatic maintenance of FAT in host neurons, ultimately contributing to their dysfunction and degeneration (see “Neuronal Kinases: Potential Role on Axonal Transport of Neurotropic Viruses and Pathological Implications” section).

## Entry Routes Shape The Mode of Neurotropic Virus Engagement in Fat

**Figure 2:**
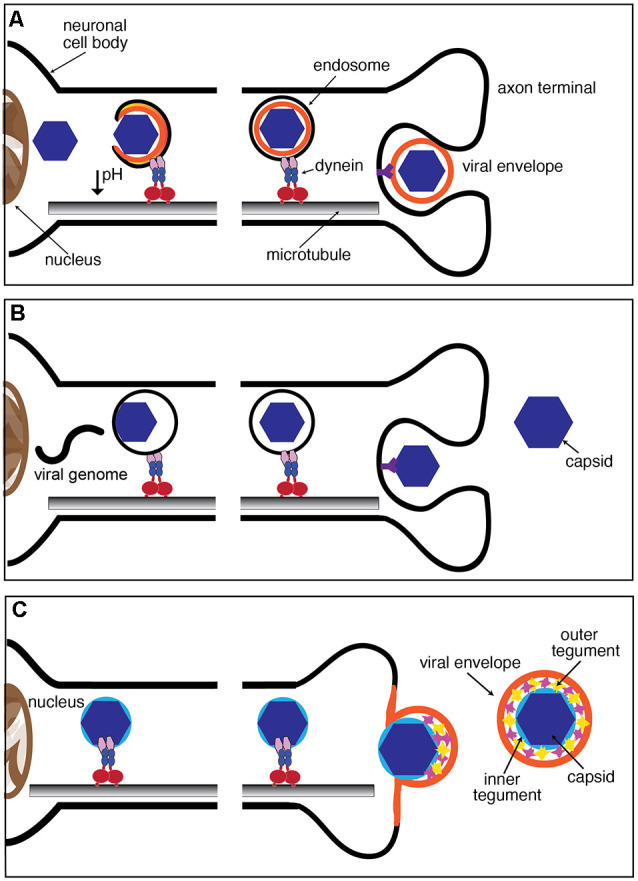
Mechanisms of neuronal entry by viruses. **(A,B)** Both enveloped and non-enveloped viruses can be internalized through the endocytic pathway following receptor binding. Endosomes are transported from the axon terminal region to the soma as part of the endogenous FAT pathway. Release of the virion from the endosome occurs after or during retrograde axonal transport and in some cases can be triggered by a decrease in pH at the endosomal lumen. **(C)** Enveloped viruses, including alphaherpesviruses, can fuse with the plasma membrane, resulting in the release of the capsid and tegument proteins to the cytoplasm. This fusion event results in the loss of the viral envelope prior to transport to the nucleus. Once internalized, cytoplasmic virions may directly recruit components of the dynein motor complex or associate with endogenous host organelles to engage in retrograde axonal transport to the soma.

In this section, we briefly discuss some well-established strategies utilized by viruses to invade neurons (Figure 2). One strategy, employed by both enveloped and non-enveloped viruses, involves the internalization of virions within endocytic vesicles at axonal termini, followed by the release of viral capsids or genomes upon arrival to the neuronal soma. Another strategy, used by enveloped viruses, involves fusion with the plasma membrane, which instead results in the release of viral capsids and capsid-associated proteins into the axonal cytoplasm. As discussed in “Engagement of Virions With Specific Components of the Host’s Fast Axonal Transport Machinery” section, these strategies set the stage for subsequent interaction of viruses with specific components of the host FAT machinery.

### Hijacking Endocytic Processes to Invade Neurons: Neurotropic Viruses as Luminal Endosomal Cargoes

Endocytosis is a cellular process used by all cells to internalize extracellular materials and facilitate the homeostatic maintenance of lipids and proteins associated with the plasma membrane (Cossart and Helenius, 2014; Elkin et al., 2016). Endocytosis is a highly regulated cellular process in neurons, particularly at axonal termini, where it contributes to the maturation and sustained recycling of synaptic vesicles (Pigino et al., 2012). Following binding to specific receptors in the neuronal plasma membrane, numerous neurotropic viruses exploit the host endocytic machinery for entry. Rabies virus (RV), adenovirus (AV), and dengue virus (DV) are examples of viruses that enter neurons through a specialized mode of endocytosis involving the scaffolding protein *clathrin* (Salinas et al., 2009; Piccinotti and Whelan, 2016; Ho et al., 2017). Clathrin promotes membrane curvature by polymerizing around the cytoplasmic face of invaginated membranes. Concurrently, clathrin also recruits accessory proteins needed for eventual fission of newly formed endocytic vesicles, including the GTPase dynamin (reviewed in Popova et al., 2013). Whether RV, AV, and DV are all internalized in endocytic vesicles of the same biochemical composition remains unknown, but recent studies revealed that RV is internalized within signaling endosomes containing the Trk receptor p75NTR (Gluska et al., 2014). As newly formed endosomes undergo their journey on retrograde FAT, they concurrently undergo maturation into *late endosomes*, a process involving acidification of their lumen (Parton et al., 1992; Overly et al., 1995; Gluska et al., 2014). As these organelles finally approach the neuronal soma, the reduction in luminal pH causes some enveloped neurotropic viruses, including RV, DV, and West Nile virus (WNV) to fuse with the endosomal membrane, resulting in the release of viral capsids and genome to the cytosol (Krishnan et al., 2007; Nelson et al., 2009; Huotari and Helenius, 2011; Piccinotti and Whelan, 2016; Ho et al., 2017; Figure 2A).

Poliovirus (PV) and Japanese encephalitis virus (JEV) are unique in that they enter cells through clathrin-independent endocytosis (Brandenburg et al., 2007; Coyne et al., 2007; Bergelson, 2008; Kalia et al., 2013). PV is a non-enveloped virus and therefore, cannot fuse with the endosome to release the capsid to the cytosol. Instead, the viral genome is likely released to the cytoplasm directly from the endocytic vesicle (Figure 2B). Events that trigger the release of the PV genome from the capsid remain unknown, although it has been established that it does not involve a decrease in pH at the endocytic vesicle lumen (Perez and Carrasco, 1993; Brandenburg et al., 2007). Intact PV virions were observed along axons of infected neurons, suggesting that this virus delays the release of its genome until the late endosomes reach the neuronal soma (Ohka et al., 1998). Notably, this is distinct from infection of non-neuronal cells, where PV genome release occurs in close proximity to the plasma membrane (Brandenburg et al., 2007).

Much remains unknown about mechanisms that coordinate the initial recruitment of CDyn to endocytic vesicles and its activation as a processive motor (Canty and Yildiz, 2020). As discussed in “Fast Axonal Transport, A Cellular Process Powered by Microtubule-Based Motor Proteins” section, experimental evidence has emerged suggesting these events may involve phosphorylation of DIC subunits (Pfister, 2015), but alternative mechanisms involving interactions between other CDyn subunits with a wide variety of adaptor proteins have also been proposed (Canty and Yildiz, 2020). As discussed in “Neuronal Kinases: Potential Role on Axonal Transport of Neurotropic Viruses and Pathological Implications” section, some neurotropic viruses reportedly activate various host kinases following the neuronal invasion, including some involved in the phosphorylation of selected CDyn subunits and putative adaptor proteins. Based on these precedents, it seems conceivable that localized activation osf such kinases at axonal terminals could promote the recruitment of CDyn to endocytic vesicles containing virions as cargo. Consistent with this possibility, several independent studies documented anti-viral effects of various pharmacological kinase inhibitors, but whether these inhibitors impact FAT of viruses has not yet been evaluated (Ye et al., 2013; Manjunatha et al., 2017).

PV uses the type I transmembrane glycoprotein CD155 as a receptor (Mendelsohn et al., 1989). Interestingly, some experimental evidence suggested that retrograde FAT of PV, which likely travels as a luminal vesicle cargo, may involve a direct interaction between CD155 and CDyn. In support, the cytoplasmic domain of CD155 was found to interact with the DLC subunit Tctex-1 in a yeast two-hybrid system assay, as well as in co-immunoprecipitation experiments involving overexpression of these proteins in non-neuronal cells (Mueller et al., 2002; Ohka et al., 2004). These results raised the possibility that binding of CD155 to Tctex somehow facilitates the recruitment of CDyn to PV-containing endosomes, but such a mechanism has yet to be evaluated *in vivo*.

### Fusion With the Plasma Membrane: The Case of Alphaherpesviruses

Alphaherpesvirus virions consist of a protein capsid surrounded by a dense matrix of proteins (referred to as the tegument) and a lipid envelope studded with viral glycoproteins (Zhou et al., 1999; Huet et al., 2016). Early images of herpes simplex virus type 1 (HSV-1) virions fusing with the plasma membrane of cultured sensory neurons suggested that this highly neurotropic virus does not enter neurons through an endocytic entry vesicle (Lycke et al., 1988; Figure 2C). Transmission immunoelectron microscopy of HSV-1 infection of rat dorsal root ganglia (DRG) neurons and primary human DRG axons demonstrated loss of the outer tegument proteins following virion fusion with the plasma membrane in axonal terminals (Aggarwal et al., 2012). Live cell imaging studies on the related neuroinvasive alphaherpesvirus, pseudorabies virus (PRV) revealed that most viral capsids undergoing retrograde FAT are not enveloped, a finding consistent with envelope fusion with the plasma membrane as the main route of entry (Luxton et al., 2005; Antinone and Smith, 2010). However, recent data adds complexity to the proposed model of HSV-1 neuronal entry. Pharmacological inhibition of dynamin, a GTPase with an essential role in membrane fission during endocytosis (Ferguson and De Camilli, 2012), reduced HSV-1 entry and transport of capsids to the nucleus in both SK-N-SH human neuroblastoma cells and human fetal cortical neurons (Mues et al., 2015). In contrast, observations on HSV-1 infection of primary mouse hippocampal neurons indicated that infection was dynamin-independent (Rahn et al., 2011). Whether these seemingly conflicting findings resulted from different experimental conditions including differences in species, cell type, or HSV-1 strain remains unclear.

## Engagement of Virions with Specific Components of The Host’S Fast Axonal Transport Machinery

The engagement of neurotropic viruses in either retrograde or anterograde FAT has often been assumed to involve specific interactions among viral and host motor proteins. However, a possibility exists that certain characteristics of viruses unrelated to peptide sequences, such as overall charge and hydrophobicity, facilitate interactions with molecular components on the surface of retrogradely moving MBOs (Leopold and Pfister, 2006). Regardless, the specific invasion strategy followed by each virus is expected to shape their mode of engagement. For example, the fusion of enveloped alphaherpesvirus to the neuronal axolemma results in exposure of inner tegument proteins to the neuronal cytosol, conceivably allowing an interaction of viral proteins with motors or with other components on the surface of MBOs (Figure 2C). In contrast, transport of virions in the lumen of endocytic vesicles would presumably restrict the access of viral proteins to molecular motors in the axonal cytosol.

**Figure 3:**
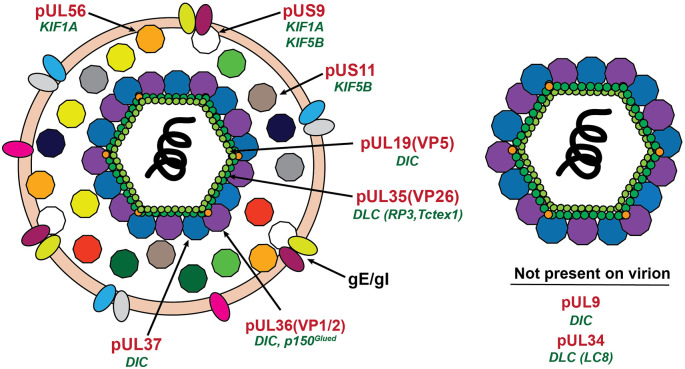
Location of proposed motor binding proteins in the alphaherpesvirus virion. The mature extracellular virion is shown on the left. The post-fusion (see Figure 2C) virion is shown on the right. Proteins with reported interactions with microtubule motor proteins are labeled in red with the motor protein subunit listed in green below.

Although a molecular basis underlying the engagement of specific neurotropic viruses on FAT remains to be established, numerous interactions between viral proteins and microtubule-based motor proteins have been reported in the published literature. In this section, we describe some of these interactions, providing a cautionary note on the interpretation of results from *in vitro* experiments that led to their identification. Our discussion of this topic features a heavy focus on alphaherpesviruses, the main neurotropic virus for which specific viral proteins have been shown to affect viral FAT (Table 1; Figure 3).

**Table 1:** Reported interactions between alpha herpes virus proteins and microtubule-based motor proteins.

**Viral protein**	Location on mature virion	Virus used in interaction studies	Motor protein subunit	Viral FAT data
**pUL36 (VP1/2)**	Inner tegument	PRV (Zaichick et al., 2013)	DIC, p150^Glued^	**PRV**: Reduced transport velocity in the absence of dynactin binding domain (Zaichick et al., 2013) **HSV-1**: Reduced capsid motility in the absence of pUL36 (Buch et al., 2017)
**pUL37**	Inner tegument	HSV-1 (Musarrat et al., 2021)	DIC	**HSV-1**: Reduced capsid motility in the absence of pUL37 (Buch et al., 2017)
**pUL34**	Not present	HSV-1 (Ye et al., 2000)	DIC	Not Assayed
**pUL9**	Not present	HSV-1 (Martínez-Moreno et al., 2003)	DLC(LC8)	Not Assayed
**VP11/12**	Outer tegument	HSV-1 (Douglas et al., 2004)	DLC (RP3, Tctex1)	**PRV**: No change in viral transport velocity in the absence of VP11/12 (Antinone et al., 2006)
**pUL35 (VP26)**	Capsid	HSV-1 (Douglas et al., 2004; Apcarian et al., 2010)	DLC (RP3, Tctex1)	**PRV**: No change in viral transport velocity in the absence of VP26 (Antinone et al., 2006) **HSV-1**: MT based Transport to nucleus observed in the absence of VP26 (Döhner et al., 2006)
**pUL19 (VP5)**	Capsid	HSV-1 (Musarrat et al., 2021)	p150^ Glued^ p62	Not Assayed
**pUS11**	Outer tegument	HSV-1 (Diefenbach et al., 2002)	KHC (KIF5B)	Not Assayed
**pUL56**	Outer tegument	HSV-2 (Koshizuka et al., 2005)	KIF1A	**PRV**: No change in viral transport velocity in the absence of pUL56 (Daniel et al., 2016)
**pUS9**	Envelope	PRV (Kramer et al., 2012) HSV-1 (Diefenbach et al., 2016)	KHC (KIF5B), KIF1A	**HSV-1**: Mutation of kinesin binding domain caused reduction in number of particles in axons (Diefenbach et al., 2016) **PRV**: No change in viral transport velocity in the absence of pUS9 (Daniel et al., 2015)

### Interactions Between Alphaherpesvirus Proteins and Cytoplasmic Dynein

Compelling biochemical experiments identified inner tegument proteins as viral components involved in retrograde FAT of HSV-1 through interactions with host motor proteins. In these experiments, both the envelope and outer tegument proteins were removed from purified HSV-1 virions using a non-ionic detergent and KCl (Radtke et al., 2010). This treatment resulted in “stripped” viral particles with exposed inner tegument proteins including pUL36 (VP1/2), pUL37, and pUS3. The authors then compared the ability of “stripped” viral particles and HSV-1 nuclear B and C capsids, which lack both inner and outer tegument proteins, to interact with motor proteins following incubation with either cytosolic pig brain lysates or with purified CDyn and dynactin (Radtke et al., 2010). Following centrifugation, the resulting pellet fractions were analyzed by immunoblotting. Detectable amounts of DIC, DLIC, and the dynactin subunit p150^Glued^ were found to co-precipitate with “stripped” viral particles, but not with nuclear capsids, suggesting that inner tegument proteins may associate with native CDyn. Consistent with these findings, HSV-1 capsids with exposed inner tegument proteins showed robust movement along axons of cultured sensory neurons, whereas capsids lacking both inner and outer tegument proteins did not (Wolfstein et al., 2006). Intriguingly, KHC subunits of conventional kinesin, and KIF3A subunits of the anterograde motor kinesin-2 also co-precipitated with “stripped” viral particles, but not with nuclear C capsids (Radtke et al., 2010). This data raised the possibility that inner tegument proteins might recruit both retrograde and anterograde host motor proteins. Short, transient bursts of anterograde motion observed during net retrograde FAT to the nucleus appear consistent with the hypothesis that both motors associate with incoming virions, but why or how retrograde FAT would prevail overall is unclear (Smith et al., 2004; Antinone and Smith, 2010; Scherer et al., 2020).

The case for inner tegument proteins being essential mediators of alphaherpesvirus retrograde FAT was strengthened by live-cell imaging data showing that the inner tegument proteins pUL36 and pUL37 remain associated with capsids during retrograde FAT (Luxton et al., 2005; Coller et al., 2007; Antinone and Smith, 2010; Cardone et al., 2012). Both pUL36 and pUL37 were shown to be essential for intracellular capsid motility in epithelial cells and cultured sensory neurons (Buch et al., 2017). When used as bait in pull-down assays, over-expressed PRV pUL36 was found to co-precipitate with DIC subunits and with the dynactin subunit p150^Glued^ from epithelial cell lysates (Zaichick et al., 2013). However, whether these subunits interacted with pUL36 directly or indirectly was not addressed. As the presence of DHC subunits in pUL36 co-precipitates was not evaluated, whether pUL36 interacts with the CDyn holoenzyme also remains unclear. In a more physiological setting, mutations in the pUL36 domains implicated in binding to dynactin impaired PRV retrograde transport in cultured neurons, while also reducing the kinetics of PRV neuroinvasion *in vivo* (Zaichick et al., 2013). These data strongly support the role of pUL36 in the engagement of PRV in retrograde FAT. Recent studies demonstrated an interaction between pUL37 and DIC (Musarrat et al., 2021). This interaction was demonstrated by immunoprecipitation experiments using HSV-1 infected SK-N-SH cells (Musarrat et al., 2021). Independent studies showed that mutations in an exposed region of the UL37 tegument protein prevented sustained retrograde FAT of PRV and HSV-1 virions in cultured sensory neurons (Richards et al., 2017), suggesting a role for pUL37 retrograde FAT of internalized HSV-1 and PRV virions, however, it is not known if these mutations disrupted potential pUL37/DIC interactions.

Interactions between CDyn and alphaherpesvirus proteins outside of the inner tegument have also been reported. Immunoprecipitation studies using lysates from HSV-1-infected epithelial cells or purified viral proteins as starting material suggested an interaction between DIC subunits and the HSV-1 tegument protein pUL34 (Ye et al., 2000). In addition, synthetic peptides corresponding to the HSV-1 helicase pUL9 interacted with a recombinant form of the DLC subunit LC8 *in vitro* (Martínez-Moreno et al., 2003). However, neither pUL9 nor pUL34 is present in mature virions, making it unlikely that these interactions could facilitate retrograde FAT of HSV-1 virions *in vivo* (Fuchs et al., 2002; Loret et al., 2008).

Studies involving yeast-two hybrid screens and *in vitro* pull-down assays documented an interaction between the HSV-1 capsid protein pUL35 (VP26), the tegument protein pUL46 (VP11/12), and the DLC subunits RP3 and Tctex1 (Douglas et al., 2004). The interaction between the VP26 and DLC subunits was further mapped to the N-terminal half of VP26 (Douglas et al., 2004; Apcarian et al., 2010). Extending these findings, imaging experiments involving microinjection of HSV-1 capsids into HepG2 epithelial cells showed reduced accumulation of capsids lacking VP26 at the nuclear rim (Douglas et al., 2004). However, subsequent studies showed that HSV-1 capsids containing VP26 failed to bind to DLC subunits in the absence of inner tegument proteins (Radtke et al., 2010). Further, independent studies using Vero cells and isolated sensory neurons reported efficient retrograde FAT of both HSV-1 and PRV virions lacking VP26 (Antinone et al., 2006; Döhner et al., 2006). Collectively, these data strongly suggest that VP26 does not play a predominant role in retrograde FAT of HSV-1. Immunoprecipitation studies using HSV-1 infected SK-N-SH cells reported an interaction between the HSV-1 capsid protein, VP5, and the dynactin proteins DCTN1/p150 and DCTN4/p62 (Musarrat et al., 2021). Thus far, there is no functional data supporting a role for VP5 in promoting retrograde transport of HSV-1 virions.

### Interactions Between Alphaherpesvirus Proteins and Kinesins

Following their replication and acquisition of an envelope membrane in the neuronal soma, newly formed alphaherpesvirus virions need to engage in anterograde FAT to reach their site of egression at axonal terminals (Taylor and Enquist, 2015). Specific mechanisms underlying anterograde FAT of these viruses remain a matter of debate, but three viral proteins (pUS11, pUL56, and pUS9) have been reported to interact with kinesin-related motor proteins (see Table 1). It should be noted that we are limiting our discussion to alphaherpesvirus proteins, for which data suggest a direct interaction with specific members of the kinesin superfamily of proteins. Several studies implicated the glycoproteins gE/gI in anterograde FAT of HSV-1 and PRV (Tirabassi et al., 1997; Husak et al., 2000; McGraw et al., 2009; Kratchmarov et al., 2013). To the best of our knowledge, no studies have shown a direct interaction between gE/gI and molecular motor proteins. Therefore, these proteins will be discussed only in the context of their cooperation with viral proteins that have already been proposed to interact with molecular motors.

Using lysates prepared from HEp-2 cells infected with HSV-1 as starting material, the tegument protein pUS11 was found to co-precipitate with bacterially expressed, recombinant KIF5B (a KHC subunit isoform) in pull-down assays (Diefenbach et al., 2002), but whether this protein behaves as conventional kinesin holoenzyme is unclear (see “Experimental Approaches to Identify and Address Viral/Motor Protein Interactions: Limitations and Cautionary Notes” section below). Thus, whether pUS11 interacts with endogenous KIF5B, and whether this interaction affects anterograde FAT of HSV-1 *in vivo* remains to be determined. Given these limitations, the physiological relevance of pUS11-KHC interactions remains largely unknown.

The membrane-associated tegument protein pUL56 of HSV-2 was proposed to be an anterograde FAT effector based on data from yeast two-hybrid system and pull-down assays showing an interaction with the kinesin-related motor protein KIF1A (Koshizuka et al., 2005). Deletion of pUL56 in HSV-1 decreased pathogenicity in multiple animal models of infection (Rösen-Wolff et al., 1991), but it is unclear whether this effect resulted from abolishing an interaction with KIF1A. Recent studies showed that silencing of KIF1A in CAD cells, a mouse neuronal cell line, did not affect anterograde axonal transport of HSV-1. Instead, such transport was reliant on the conventional kinesin heavy chain subunits KIF5A, KIF5B, and KIF5C (DuRaine et al., 2018). In addition, HSV-1 capsids colocalized with GFP-tagged KIF5C but not GFP-tagged KIF1A in infected CAD cells (DuRaine et al., 2018). In contrast, data on HSV-1 infection of primary mouse sensory neurons showed colocalization between HSV-1 capsids and KIF1A in axons (Scherer et al., 2020). These seemingly conflicting results suggest that either the species or type of neuron (peripheral or central nervous system) might impact the specific motor protein used for anterograde FAT. Investigation of the role of pUL56 in anterograde FAT of PRV showed that loss of pUL56 resulted in decreased pathogenicity in a mouse model of infection, however, pUL56 deletion had no impact on anterograde FAT of PRV in cultured sensory neurons or in a rat eye model of infection (Daniel et al., 2016).

When over-expressed in epithelial cells, the HSV-1 US9 tegument protein (pUS9) co-precipitated with endogenous KIF5B in pull-down assays (Diefenbach et al., 2016). Deletion experiments mapped this interaction to the basic domain of pUS9 and the carboxy-terminal domain of KIF5B. Although pUS9 reportedly associates with HSV-1 capsids moving anterogradely towards axon terminals, the role of this protein during anterograde transport of HSV-1 remains controversial (Polcicova et al., 2005; LaVail et al., 2007; Snyder et al., 2008). Infection with HSV-1 virions lacking pUS9 or with mutations in the KIF5B binding region results in reduced numbers of particles moving anterogradely in cultured sensory neurons and human SK-N-SH neuroblastoma cells, compared to infection with wild-type HSV-1 (Snyder et al., 2008; McGraw et al., 2009; Diefenbach et al., 2016). In addition, mutation of the KIF5B-binding domain reduced zosteriform disease in a mouse model of HSV-1 infection, which could be due to a reduction in anterograde HSV-1 spread (Diefenbach et al., 2016). However, HSV-1 virions lacking pUS9 retain the ability to spread anterogradely to the brain following retinal infection or to the spinal cord following flank inoculation, albeit with reduced efficiency (McGraw et al., 2009). Collectively, this data suggests that while pUS9 may contribute to anterograde FAT of HSV-1, it is not essential and likely facilitates this process in cooperation with other viral proteins *in vivo* (McGraw et al., 2009).

Independently, mass spectrometry studies reported an interaction between PRV pUS9 and KIF1A (Kramer et al., 2012). Over-expression of a dominant negative KIF1A variant reduced the number of PRV capsids undergoing anterograde FAT in axons of cultured rat SCG neurons, but whether this effect resulted from disrupting pUS9 interaction with endogenous KIF1A was not determined (Kramer et al., 2012). Recent data suggest that during PRV infection gE/gI forms a complex that recruits KIF1A in a pUS9-dependent manner (Diwaker et al., 2020; Scherer et al., 2020). *In vivo* experiments addressing the role of pUS9 on PRV anterograde FAT yielded complicated results. Early studies using PRV in a rat model of infection demonstrated that virions lacking pUS9 failed to spread anterogradely within the visual system (Brideau et al., 2000). More recently, live-cell imaging experiments demonstrated that deletion of pUS9 in PRV virions reduced the overall number of virions sorted to the axon, yet their anterograde transport velocity remained unaffected (Daniel et al., 2015). In conclusion, the available experimental data support a role for pUS9 during anterograde FAT, but whether such role involves direct interactions with KIF5B subunits of conventional kinesin or with KIF1A remains unclear. Interestingly, recent data showed that during PRV infection of neuronal cells the US9/gE/gI protein complex results in accelerated proteasomal degradation of KIF1A proteins in axons (Huang et al., 2020). This data highlights how a viral infection has the potential to alter endogenous FAT through depletion of motor proteins.

### Experimental Approaches to Identify and Address Viral/Motor Protein Interactions: Limitations and Cautionary Notes

As described above, various interactions between viral proteins and selected motor protein subunits have been identified using pull-down assays, yeast-two hybrid, and other *in vitro* experimental systems suggested. However, whether such interactions extend to functional motor protein holoenzymes remains to be addressed in most cases. We caution on the interpretation of data involving overexpression of motor protein subunits *in isolation*, as their folding state often differs significantly from that in native motor holoenzymes. Indeed, KHC, KLC, and DIC subunits all feature prominent hydrophobic patches that render them susceptible to non-specific interactions when expressed in isolation. For example, a high-affinity interaction was reported between recombinant KLC1 and the Alzheimer’s disease-related amyloid precursor protein (APP), leading to the claim that APP might act as an adaptor protein linking conventional kinesin to MBO cargoes (Kamal et al., 2000). However, bacterially expressed KLC1 was later found to interact non-specifically with a wide variety of unrelated proteins, including GFP, and an association between APP and conventional kinesin could not be demonstrated using native brain lysates as starting material or *in vivo* (Lazarov et al., 2005). In a manner analogous to KLCs, a wide variety of unrelated proteins, including huntingtin and snapin were found to interact with overexpressed DICs in pull-down assays (Caviston et al., 2007; Tammineni et al., 2017). However, an interaction of huntingtin with DICs could not be confirmed by immunoprecipitation using brain lysates as starting material (Morfini et al., 2009b). The unique expression pattern of motor protein subunits is another factor limiting the relevance of data obtained from pull-down assays, as non-neuronal cell lines used to overexpress viral proteins typically express a small subset of these subunits (Kuta et al., 2010). For example, DIC1 subunits, which are involved in retrograde FAT of signaling endosomes in neurons, are not expressed in non-neuronal cells (Mitchell et al., 2012). Collectively, these issues raise a cautionary note on the physiological relevance interactions between viral proteins and motor proteins identified *in vitro* (Dodding and Way, 2011). To ensure such interactions indeed extend to functional motor holoenzymes, experimental systems featuring native motor proteins, rather than over-expressed subunits in isolation, should be preferred. More importantly, *in vivo* experiments are needed to determine whether disrupting putative viral/motor subunit interaction indeed affects the engagement of viruses in either anterograde or retrograde FAT (Merino-Gracia et al., 2011).

## Neuronal Kinases: Potential Role on Axonal Transport of Neurotropic Viruses and Pathological Implications

As discussed in “Fast Axonal Transport, A Cellular Process Powered by Microtubule-Based Motor Proteins” section, the sustained functionality of specialized neuronal subcompartments (i.e., pre-synapses and nodes of Ranvier) critically depends on the regulated trafficking, delivery, and recycling of MBOs (Morfini et al., 2016). FAT is therefore regarded as an essential cellular process sustaining neuronal connectivity and survival (Britt et al., 2016). Supporting this notion, mutations in genes that encode CDyn and conventional kinesin subunits have been linked to inheritable neurological conditions involving progressive dysfunction and degeneration of specific neuronal populations (reviewed in Lipka et al., 2013; Kalantari and Filges, 2020). More recently, the identification of kinase pathways involved in the regulation of FAT provided novel insights into mechanisms by which neuropathogenic proteins might promote neuronal dysfunction. Specifically, mutant proteins linked to familial forms of neurodegenerative diseases including huntingtin, superoxide dismutase 1, tau, and spastin were all found to cause FAT deficits through a mechanism involving deregulation of selected kinase pathways (reviewed in Morfini et al., 2009a; Brady and Morfini, 2010). Relevant to the topics discussed in this review, neurotropic viruses have been widely shown to modulate the activity of host kinases (Zachos et al., 1999; Piacentini et al., 2015; Bonjardim, 2017; Besson et al., 2019), including some involved in the regulation of FAT. A thorough description of these findings is beyond the scope of this review, but some relevant examples are provided below. Based on these precedents, it is conceivable that aberrant activation of kinases might contribute to the various neuronal pathologies associated with viral infection (Mitchell et al., 2012).

### Virus-Induced Activation of Kinases Involved in the Regulation of Fast Axonal Transport

Infection of neurons by JEV, RV, and HSV-1 increases activation of ERK1/2 and PKC (Park and Baines, 2006; Yang et al., 2010; Ye et al., 2013; Manjunatha et al., 2017), kinases with an established role in the activation of CDyn-based retrograde FAT (Morfini et al., 2007; Mitchell et al., 2012). As speculated in see “Entry Routes Shape the Mode of Neurotropic Virus Engagement in FAT” section, neurotropic viruses could increase activation of these kinases during infection to promote retrograde FAT of endocytic MBOs, a fraction of which will contain incoming viral particles. Supporting this hypothesis, pharmacological inhibition of ERK1/2 (Manjunatha et al., 2017) and PKC (Lama et al., 2019) reduced RV replication in the brain in a mouse model of infection, although the pleiotropic actions of ERK1/2 make it difficult to determine whether this is an effect on FAT or another cellular process. Beyond the neurotropic viruses covered in this review, other viruses have been shown to interact with cellular kinases to promote transport. Among those, human immunodeficiency virus (HIV) was found to interact with MARK2/PAR-1 kinase and promote phosphorylation of the putative KIF5 adaptor protein FEZ1 (Malikov and Naghavi, 2017), an event linked to HIV capsid transport and uncoating (Malikov et al., 2015; Malikov and Naghavi, 2017).

Aberrant modulation of kinases involved in the regulation of retrograde FAT may also promote pathological effects associated with abnormal turnover of MBOs and altered phosphorylation of critical substrates in axonal terminals. For example, Borna disease virus (BDV) has been shown to disrupt synaptic function through a mechanism involving PKC inhibition and reduced phosphorylation of synaptic protein substrates (i.e., MARCKS and SNAP25; Volmer et al., 2006). The pathogenic effects of BDV were mapped to the viral phosphoprotein P (Prat et al., 2009). Expanding on these findings, ectopic expression of phosphoprotein P in the mouse hippocampus induced a marked impairment in long–term memory that was associated with reduced phosphorylation of synaptic PKC substrates (Bétourné et al., 2018). Whether the alteration in PKC activity associated with BDV infection affects retrograde FAT of endogenous MBOs has yet to be evaluated, but evidence from experiments in the squid giant synapse preparation appears consistent with such a possibility (Serulle et al., 2007). Regardless, these experimental precedents demonstrate that localized changes in kinase activity triggered by specific viral proteins suffice to impact neuronal connectivity.

The ability of viral components to affect cellular signaling pathways may also relate to other aspects of viral pathogenesis. For example, viral proteins shed from infected cells may also promote aberrant activation of kinases that in turn impact FAT. The HIV protein gp120 is shed from infected macrophages and undergoes internalization in neurons (Mocchetti et al., 2012; Berth et al., 2015). Toxic actions of intracellular gp120 remained unknown, but recent studies in the isolated squid axoplasm preparation revealed a marked inhibition of both anterograde and retrograde FAT induced by picomolar levels of this protein (Berth et al., 2016). Pharmacological experiments revealed these toxic effects involved activation of Tak-1, a MAP3K upstream of p38 and JNK kinases (Gallo and Johnson, 2002). Increased p38 activation in response to gp120 treatment was also demonstrated in primary cortical neurons (Hu et al., 2005; Medders et al., 2010; Berth et al., 2015). Extending these findings to other HIV proteins, the addition of recombinant Tat protein to cultured striatal neurons increased activation of GSK-3, as well as p38 and JNK MAPKs (Singh et al., 2005). Significantly, MAPKs are also activated by a wide variety of neurotropic viruses including alphaherpesviruses, enteroviruses, and flaviviruses (Perkins et al., 2002; Hargett et al., 2005; Wong et al., 2005; He et al., 2017). In sum, deregulation of selected kinases by specific viral proteins may negatively impact FAT and other phosphorylation-dependent cellular processes sustaining neuronal function.

### Conclusions and Therapeutic Implications

Both the symptoms and pathology associated with neuronal infection have been extensively described (Berth et al., 2009). Unfortunately, little is known about pathogenic mechanisms by which viruses induce dysfunction and degeneration of neurons. The identification of specific viral and host effector proteins mediating the engagement of neurotropic viruses in FAT may reveal important clues on such mechanisms and provide potential targets for the development of novel anti-viral interventions. For example, some neurotropic viruses could affect FAT in the host cell by directly sequestering microtubule-based motors or through modulation of kinases involved in the regulation of this cellular process. Under the first scenario, therapeutic efforts aimed to reduce viral infection might require knowledge of specific viral proteins interacting with microtubule-based motors, since targeting the motors themselves would be expected to cause adverse effects on neurons. In the second scenario, therapeutic treatments would require knowledge of specific kinases activated by neuroinvasive viruses and/or viral proteins.

Given the number of kinases activated at different stages of the viral cycle and the complexity and crosstalk among kinase pathways, the identification of pathologically relevant ones may represent a major challenge for investigators (Besson et al., 2019). Nevertheless, a message of hope is provided by the successful development of highly specific, brain-permeable kinase inhibitors (Maphis et al., 2016), and increasing experimental evidence confirming a role of host kinases on the neuronal pathology triggered by some viruses (Beckham et al., 2007). These recent developments support the notion that pathologically relevant kinases can be targeted to restore their activities to normal levels (Cuny, 2009; Munoz and Ammit, 2010). Although more experiments are needed to lend support to this hypothesis, we feel that anti-viral therapies targeting aberrantly active kinases have the potential to restore FAT abnormalities and prevent, at least in part, pathological phenotypes associated with such abnormalities during viral infection (Brady and Morfini, 2017).

## Author Contributions

GM, AR, and SHB conceived the manuscript and generated the first drafts. All authors contributed to the article and approved the submitted version.

## Conflict of Interest

The authors declare that the research was conducted in the absence of any commercial or financial relationships that could be construed as a potential conflict of interest.
